# COVID-19 Vaccination Perspectives and Illnesses Among Law Enforcement Officers, Firefighters, and Other First Responders in the US, January to September 2021

**DOI:** 10.1001/jamanetworkopen.2022.22640

**Published:** 2022-07-19

**Authors:** Alberto J. Caban-Martinez, Manjusha Gaglani, Lauren E. W. Olsho, Lauren Grant, Natasha Schaefer-Solle, Mark G. Thompson, Jefferey L. Burgess

**Affiliations:** 1 Department of Public Health Sciences, Leonard M. Miller School of Medicine, University of Miami, Miami, Florida; 2 Baylor Scott and White Health, Texas A&M University College of Medicine, Temple; 3 Abt Associates, Rockville, Maryland; 4 Centers for Disease Control and Prevention COVID-19 Response Team, Atlanta, Georgia; 5 Mel and Enid Zuckerman College of Public Health, University of Arizona, Tucson

## Abstract

This cohort study assesses attitudes toward COVID-19 vaccination and illness burden among vaccinated and unvaccinated law enforcement officers, firefighters, and other first responders in the US.

## Introduction

Law enforcement officers, firefighters, and other first responders are at increased risk of SARS-CoV-2 infection compared with health care personnel^1^ but have relatively low COVID-19 vaccine uptake.^2^ COVID-19 was the leading cause of line-of-duty deaths among US law enforcement officers in 2021 (323 of 482 deaths [67%]).^3^ Using data from the Arizona HEROES (Healthcare, Emergency Response, and Other Essential Workers Study) and RECOVER (Research on the Epidemiology of SARS-CoV-2 in Essential Response Personnel) cohorts, we assessed attitudes toward COVID-19 vaccination and illness burden among vaccinated and unvaccinated first responders.

## Methods

The HEROES and RECOVER studies are cohorts of first responders and other essential workers with a shared data collection protocol^4,5^ (eMethods in the Supplement). Eligible participants were enrolled in Florida, Minnesota, Oregon, Texas, Utah, and Arizona; were 18 to 85 years of age; and worked at least 20 hours per week in occupations involving direct contact (<3 feet) with others. Information on participant race and ethnicity was self-reported on the enrollment survey to understand how attitudes and vaccination practices differ in the US first responder workforce that is traditionally non-Hispanic White.

From January 1 to September 30, 2021, participants contributed weekly nasal specimens using home-based kits and reported COVID-19–like symptoms via text message. Respiratory specimens were tested using reverse transcriptase–polymerase chain reaction assay for SARS-CoV-2. Participant characteristics were collected at enrollment; COVID-19–related duration of illness and missed work were assessed at the beginning and end of each illness. Vaccine knowledge, attitudes, and practices were assessed in quarterly follow-up surveys. Participants were considered fully vaccinated 14 days after receipt of all recommended primary vaccine doses. All participants provided written informed consent. Study protocols were approved by the institutional review boards at participating sites. This study followed the STROBE reporting guideline.

Sociodemographic characteristics and attitudes toward COVID-19 vaccines were compared for unvaccinated vs fully vaccinated participants with standardized mean difference and χ^2^ and 2-sample *t* tests. Incidence rates of COVID-19 were calculated from January through September 2021 using repeated-measures Poisson regression (eMethods in the Supplement) and compared by first responder type. Statistical significance was defined as a 2-tailed *P* < .05. All analysis were conducted with SAS software, version 9.4 (SAS Institute, Inc)

## Results

Among 1415 participants, 1113 (79%) were men; 302 (21%), women; 416 (29%), Hispanic; and 1338 (95%), White. In terms of occupation, 964 were firefighters (68%); 238, law enforcement officers (17%); and 213, other first responders (15%) (Table). Mean (SD) age was 41.3 (9.7) years. Of these, 1163 participants (82%) completed an attitude survey (363 of 586 [62%] unvaccinated vs 800 of 829 [97%] vaccinated). Among the fully vaccinated, 406 (35%) said they trusted the government regarding COVID-19 vaccines; among the unvaccinated, 45 (12%) trusted the government. Unvaccinated first responders were less likely than their vaccinated counterparts to believe that COVID-19 vaccines are effective (61 [17%] vs 430 [54%], respectively) or safe (54 [15%] vs 435 [54%], respectively).

**Table.** Sociodemographic Characteristics of First Responders, Attitudes Toward COVID-19 Vaccines, and Incidence and Characteristics of Laboratory-Confirmed COVID-19 Illness, January to September 2021

Sociodemographic characteristics	Participant group^a^			Standardized mean difference^b^	*P* value^c^
	All	Unvaccinated	Fully vaccinated		
Total participants^d^	1415 (100)	586 (41)	829 (59)	NA	NA
Age, mean (SD), y	41.3 (9.7)	38.8 (9.7)	43.1 (9.4)	0.46	<.001
Sex					
Men	1113 (79)	462 (79)	651 (79)	0.01	.89
Women	302 (21)	124 (21)	178 (21)		
Race					
White	1338 (95)	554 (95)	784 (95)	0.00	.98
Other^e^	77 (5)	32 (5)	45 (5)		
Ethnicity					
Hispanic	416 (29)	190 (32)	226 (27)	0.11	.04
Non-Hispanic	999 (71)	396 (67)	603 (73)		
Cohort location					
Arizona	692 (49)	265 (45)	427 (51)	0.63	<.001
Florida	519 (37)	291 (50)	228 (27)		
Oregon	42 (3)	4 (1)	38 (5)		
Minnesota	35 (2)	2 (0.3)	33 (4)		
Texas	34 (2)	12 (2)	22 (3)		
Utah	93 (7)	12 (2)	81 (10)		
Occupation					
Firefighter	964 (68)	419 (71)	545 (66)	0.15	.03
Law enforcement officer	238 (17)	81 (14)	157 (19)		
Other^f^	213 (15)	86 (15)	127 (15)		
Attitudes toward COVID-19 vaccine					
Completed survey^g^	1163 (82)	363 (62)	800 (97)	NA	NA
Trust what government says about the vaccine	406 (35)	45 (12)	361 (45)	0.91	<.001
Believe vaccine is very or extremely effective	491 (42)	61 (17)	430 (54)	1.01	<.001
Believe vaccine is very or extremely safe	489 (42)	54 (15)	435 (54)	1.10	<.001
Laboratory-confirmed COVID-19 illness					
Total No. with COVID-19 illnesses	184	160	24	NA	NA
Before vaccine availability^h^	103 (56)	103 (100)	NA	NA	NA
During vaccine availability^i^	81 (44)	57 (70)	24 (30)	NA	NA
Laboratory-confirmed COVID-19 incidence per 1000 person-weeks (95% CI)^j^^,^^k^					
All first responders	4.0 (3.1-4.8)	9.4 (7.2-12.2)	1.7 (1.1-2.5)	NA	<.001
Firefighters	4.0 (3.0-5.3)	9.0 (6.4-12.7)	1.8 (1.1-2.8)	NA	<.001
Law enforcement officers	2.8 (1.3-5.8)	11.9 (7.0-20.1)	0.6 (0.2-2.5)	NA	<.001
Other	5.0 (3.0-8.3)	8.7 (4.6-16.4)	2.9 (1.3-6.3)	NA	.04
Duration of COVID-19 illnesses, mean (SD), d^k^	12.1 (21.7)	19.7 (16.9)	15.3 (11.7)	0.30	.25
Missed work due to COVID-19, mean (SD), h^k^	26.7 (40.0)	85.2 (49.3)	67.6 (25.8)	0.45	.19

Abbreviation: NA, not applicable.

^a^ Unless otherwise indicated, data are expressed as No. (%) of participants based on total numbers in the first row. Percentages have been rounded and may not total 100. Includes 286 participants who received 2 doses of BNT162b2 (Pfizer–BioNTech); 523, 2 doses of mRNA-1273 (Moderna); and 20, 1 dose of Ad26.COV2.S (Janssen). Seventy-one individuals who remained partially vaccinated (days 1-13 after a dose of Ad26.COV2.S or less than 14 days after dose 2 of either messenger RNA vaccine) were excluded from analysis. Unvaccinated individuals self-reported that they had not received a dose of COVID-19 vaccine in weekly surveillance messages and in electronic or telephone surveys conducted every 4 to 6 weeks.

^b^ Standardized mean or proportion difference between unvaccinated and fully vaccinated participants of more than 0.20 was considered noteworthy.

^c^ Calculated with χ^2^ tests for proportions and 2-sample *t* tests for means.

^d^ Total participants include all self-identified first responders who were fully enrolled, participated in active surveillance for COVID-19–like illness, and were documented as fully vaccinated or unvaccinated.

^e^ Includes American Indian or Alaska Native, Asian or Pacific Islander, Black or African American, other, or multiple races.

^f^ Included nonfire emergency medical services (n = 141), corrections (n = 51), and other (n = 21).

^g^ Of the 252 participants (18%) missing the attitude survey, 88 unvaccinated and 1 vaccinated participant discontinued the study before the administration of the attitude survey; the remaining 135 unvaccinated and 28 vaccinated participants failed to complete the attitude survey. Each attitude had 5 response options. Effectiveness and safety compare extremely or very with somewhat, not too, or not at all. Trust item compares strongly agree or mildly agree with neutral, mildly disagree, or strongly disagree.

^h^ Data for unvaccinated calculated as row percentage. Prior to vaccine availability refers to infections confirmed by reverse transcriptase–polymerase chain reaction in the cohort from July 9 until December 14, 2020, when COVID-19 vaccines became available in the US.

^i^ Data for unvaccinated and fully vaccinated calculated as row percentages.

^j^ Incidence rates were calculated for the period from January through September 2021 using a Poisson generalized estimating equation with occupation and vaccination status in the model and log weeks as an offset. The outcome was a SARS-CoV-2–positive COVID-19–like illness. Participants were limited to those without documentation of prior SARS-CoV-2 infection by molecular assay at the start of January 2021 and contributed to active surveillance during this period. Person-time was censored after the illness onset date of a SARS-CoV-2 infection.

^k^ Indicates among laboratory-confirmed COVID-19 illnesses during vaccine availability.

A total of 184 COVID-19 illnesses with assay-confirmed SARS-CoV-2 infection were identified among first responders. From January through September 2021, among law enforcement officers, COVID-19 incidence per 1000 person-weeks was 11.9 (95% CI, 7.0-20.1) in unvaccinated and 0.6 (95% CI, 0.2-2.5) in vaccinated individuals. Incidence was also higher among unvaccinated (9.0 [95% CI, 6.4-12.7]) vs vaccinated (1.8 [95% CI, 1.1-2.8]) firefighters. Among participants with laboratory-confirmed COVID-19, mean (SD) duration of illness (15.3 [11.7] vs 19.7 [16.9] days) and missed work (67.6 [25.8] vs 85.2 [49.3] hours) were lower among vaccinated than unvaccinated individuals, but differences were not statistically significant.

## Discussion

This cohort study found that unvaccinated first responders were more likely to develop COVID-19 and less likely to believe in the effectiveness and safety of vaccines than their vaccinated counterparts. Study limitations include a small number of COVID-19 illnesses among vaccinated individuals (n = 24), limiting precision of estimates and precluding adjustment for potential confounders. Most participants were White and from Florida and Arizona, limiting generalizability of the results. Our findings suggest that state and local governments with large numbers of unvaccinated first responders may face major workforce disruptions due to COVID-19 illness. Given the effectiveness of the COVID-19 vaccines during the public health emergency, governments should consider vaccine mandates with regular testing and alternative work assignments for unvaccinated workers. Furthermore, the low trust in government among first responders suggests a need to leverage trusted nongovernmental sources to increase vaccination rates.

## Notes

Supplement. **eMethods.** Study Design, Participants, Outcome Measures, Laboratory Methods, and Data Analysis
eReferences
