# Supplementary material for: COVID-19 Vaccination Perspectives and Illnesses Among Law Enforcement Officers, Firefighters, and Other First Responders in the US, January to September 2021
Source: JAMA Netw Open. 2022 Jul 19;5(7):e2222640. doi: 10.1001/jamanetworkopen.2022.22640 (PMC9297116; doi:10.1001/jamanetworkopen.2022.22640)
Supplement: Supplement. — eMethods. Study Design, Participants, Outcome Measures, Laboratory Methods, and Data Analysis eReferences [file jamanetwopen-e2222640-s001.pdf]

Supplementary Online Content

Caban-Martinez AJ, Gaglani M, Olsho LEW, et al. COVID-19 vaccination perspectives and illnesses among law enforcement officers, firefighters, and other first responders in the US, January to September 2021. *JAMA Netw Open*. 2022;5(7):e2222640. doi:10.1001/jamanetworkopen.2022.22640

eMethods. Study Design, Participants, Outcome Measures, Laboratory Methods, and Data Analysis

eReferences

This supplementary material has been provided by the authors to give readers additional information about their work.

© 2022 Caban-Martinez AJ et al. *JAMA Network Open*.

eMethods. Study Design, Participants, Outcome Measures, Laboratory Methods, and Data Analysis

Study Design

The HEROES-RECOVER network, initiated in July 2020, comprises two prospective cohort studies: RECOVER (Research on the Epidemiology of SARS-CoV-2 in Essential Response Personnel) and HEROES (the Arizona Healthcare, Emergency Response, and Other Essential Workers Surveillance Study) with the goal to examine SARS-CoV-2 infection and vaccine effectiveness among first responders and essential workers. Data collection across both cohorts occurred using a shared protocol. Cohort participants were enrolled in six U.S. states: Florida (Miami), Minnesota (Duluth), Oregon (Portland), Texas (Temple), Utah (Salt Lake City), and Arizona (Tucson, Phoenix, and other areas). All participants provided written informed consent. The individual protocols for the RECOVER study and the HEROES study were reviewed and approved by the institutional review boards at participating sites or under a reliance agreement.

Participants, Recruitment, and Retention

Adults aged 18 to 85 years, who worked at least 20 hours per week in occupations involving regular direct contact (within 3 feet) with others were eligible for inclusion. Eligible essential workers include health care providers (HCP), first responders, and other essential/frontline workers (EFWs). Each study site enrolled 500 to 700 participants using a 3-phase stratified recruitment approach stratified by participant sex, age group (i.e., 18-39 years and 40+ years), and occupational group (i.e., primary HCP, support HCP, first responders, and essential/frontline workers) in order to minimize potential biases and ensure a diverse population of essential workers. Recruitment and enrollment at each study site began in early August 2020 and included either a community-based recruitment approach, or recruitment through established human resource records at the study site. Site-specific methods for recruitment are available elsewhere.[1]

Participant-Reported Outcome Measures

Socio-demographic and health characteristics were collected from each participant at enrollment while the duration of illness and missed work associated with COVID-19 were collected via electronic surveys at the beginning and end of each illness episode. Vaccine intention and Knowledge, Attitudes, and Practices (KAP) questions were included in three follow-up surveys: Follow-up survey 1 (distributed late December 2020-February 2021), Follow-up survey 2 (distributed late March-May 2021), and Follow-up survey 3 (distributed July-August 2021). Participants that joined the studies during the follow-up periods received the KAP questions at the time of enrollment. The most recent survey where subjects answered KAP questions was used for unvaccinated participants. For vaccinated participants, KAP responses on the most recent survey prior to vaccination were used. The surveys included six questions to assess the KAP constructs regarding COVID-19: knowledge of SARS-CoV-2 and COVID-19 vaccines; attitudes about safety, effectiveness, trust in the government; and perceived risk of becoming ill if they were not vaccinated (Supplement Table 1). Responses to each attitude question were rated on a 5-level Likert scale indicating lowest to highest ranking. Effectiveness and safety compare "extremely or very" with "somewhat", "not too", or "not at all". Trust item compares "strongly agree" or "mildly agree" with "neutral", "mildly disagree", or "strongly disagree". Covid-19 vaccination status was reported by the participants in electronic and telephone surveys and through direct upload of images of vaccination cards. In addition, data from electronic medical records, occupational health records, or state immunization registries were reviewed at the sites in Minnesota, Oregon, Texas, and Utah.

© 2022 Caban-Martinez AJ et al. *JAMA Network Open*.

Topic	Question Text	Response Range
**Vaccine Effectiveness**	How effective do you think the COVID-19 vaccine is in preventing you from getting sick with COVID-19?	5-point Likert (1 = Not at all, 5 = Extremely effective)
**Vaccine Safety**	How safe do you think the COVID-19 vaccine is?	5-point Likert (1 = Not at all, 5 = Extremely safe)
**Trust in government**	I trust what the government says about the COVID-19 vaccine.	5-point Likert (1 = Strongly disagree, 5 = Strongly agree)

Laboratory Methods

From January to September 2021, first responders contributed nasal specimens on a weekly basis using laboratory specimen collection kits at home and reported any symptoms of COVID-like illness on a weekly basis via text message or email. Respiratory specimens were self-collected weekly, irrespective of symptoms, as well as at the onset of COVID-like illness. Specimens were tested by Reverse Transcription Polymerase Chain Reaction (RT-PCR) assay for SARS-CoV-2 at the Marshfield Clinic Research Institute (Marshfield, Wisconsin). Additional details are described elsewhere.[1]

Data Analysis Plan

Participants were considered fully vaccinated 14 days after receipt of a second mRNA COVID-19 vaccine dose or a single Johnson & Johnson's Janssen vaccine dose. Indeterminate vaccination status was defined from day of first COVID-19 vaccine dose until the participant was fully vaccinated. Unvaccinated participants had no self-reported or documented COVID-19 vaccine receipt.

Socio-demographic characteristics and attitudes toward COVID-19 vaccines were compared for all unvaccinated versus fully vaccinated first responders, based on vaccination status as of September 30th, 2021. Effect sizes were calculated using standardized means differences. P-values were calculated with chi-square tests for proportions and t-tests for means.

COVID-19 illness was defined as a positive SARS-CoV-2 PCR result with an associated report of COVID-like illness symptoms. Incidence rates of covid-19 illness were estimated during the period from January to September 2021 among first responders with no prior SARS-CoV-2 infection. Participants could contribute both unvaccinated and fully vaccinated person-time until SARS-CoV-2 detection, study withdrawal, or the end of the study period. Indeterminate vaccination time was excluded from analysis. Overall incidence was calculated as the total number of COVID-19 illnesses divided by the total person weeks, and 95% Wald confidence intervals were estimated. Incidence by occupation and vaccine status were estimated by Poisson generalized estimating equations to account for repeated participants.[2] The model included occupation, vaccine status, and their interaction as independent variables, log weeks of exposure as an offset, and participants repeating with an independent correlation structure.

Duration of illness and hours of missed work were compared among fully vaccinated and unvaccinated COVID-19 illnesses during the study observation period, January - September 2021. Vaccination status was defined as the status on the first day of the illness episode. T-tests were performed on the log transformed days of illness and hours of missed work. Statistical significance was defined as a two-tailed p-value <0.05 for all analyses. Analysis was conducted using SAS version 9.4.

© 2022 Caban-Martinez AJ et al. *JAMA Network Open*.

eReferences

1. Edwards LJ, Fowlkes AL, Wesley MG, et al. Research on the Epidemiology of SARS-CoV-2 in Essential Response Personnel (RECOVER) Study: Protocol for a Multi-site Longitudinal Cohort. JMIR Res Protoc. 2021;10(12):e31574.
2. Liang, K.-Y., & Zeger, S. L. (1986). Longitudinal Data Analysis Using Generalized Linear Models. Biometrika, 73(1), 13–22. https://doi.org/10.2307/2336267

© 2022 Caban-Martinez AJ et al. *JAMA Network Open*.
